# Association between Adherence to the Japanese Food Guide Spinning Top and Sleep Quality in College Students

**DOI:** 10.3390/nu10121996

**Published:** 2018-12-16

**Authors:** Kaori Yamamoto, Masako Ota, Ayako Minematsu, Keiko Motokawa, Yuri Yokoyama, Tomohiro Yano, Yutaka Watanabe, Takahiro Yoshizaki

**Affiliations:** 1 Graduate School of Food and Nutritional Sciences, Toyo University. 1-1-1, Izumino, Itakura-machi, Ora-gun, Gunma 374-0193, Japan; yamamoto0915093@gmail.com (K.Y.); s4c101800036@toyo.jp (A.M.); yano_t@toyo.jp (T.Y.); ywata@tmig.or.jp (Y.W.); 2 Tokyo Metropolitan Institute of Gerontology, 35-2, Sakae-cho, Itabashi-ku, Tokyo 173-0015, Japan; kikiki_1004@yahoo.co.jp (K.M.); yokoyama@tmig.or.jp (Y.Y.); 3 Department of Nutritional and Health Sciences, Faculty of Food and Nutritional Sciences, Toyo University, 1-1-1, Izumino, Itakura-machi, Ora-gun, Gunma 374-0193, Japan; 4 Department of Food and Life Sciences, Faculty of Food and Nutritional Sciences, Toyo University. 1-1-1, Izumino, Itakura-machi, Ora-gun, Gunma 374-0193, Japan

**Keywords:** food guide score, sleep quality, college student

## Abstract

This study aimed to elucidate the association between adherence to the Japanese Food Guide Spinning Top (Food Guide score) and sleep quality in Japanese college students. We conducted a cross-sectional study of 175 Japanese college students aged 19–22 years in the eastern part of Gunma Prefecture to examine the association between the Food Guide score and sleep quality. A self-administered diet history questionnaire and the Pittsburgh Sleep Quality Index were used to assess habitual dietary intake and sleep quality, respectively. In the fully adjusted model, the odds ratios for poor sleep quality in the middle and highest tertile categories of the Food Guide score were 0.50 (95% confidence interval, 0.18–1.37) and 0.30 (95% confidence interval, 0.11–0.84), respectively, compared with those in the lowest tertile category (*p* for trend = 0.033). A well-balanced diet may be associated with good sleep quality in Japanese college students.

## 1. Introduction

The daily lifestyle in Japan is diversifying. Good sleep for college students is considered to play an important role in the maintenance of mental and physical health [1,2]. The proportion of people in their 20s who sleep for less than 6 h per day is 43.9% in Japan, and this has gradually increased from 36.6% in 2003 [3]. The Lifetime Survey by the Japan Broadcasting Corporation reported that, after 1970, the number of sleeping hours on weekdays has been decreasing in Japanese students in their 20s [4]. Previous studies on the sleep quality of college students have elucidated that they have a short sleep duration [5,6], that their sleep duration changes by 1–2 h day by day, and that they experience delayed sleep phase [7], daytime sleepiness [8], and have difficulty getting to sleep [6]. Previous studies have reported that subjective sleep quality is associated with psychological well-being, such as depression and anxiety [9]. Other epidemiological studies have also shown that short sleep duration is associated with obesity [10], mortality risk [11], and cardiovascular disease [12].

Poor sleep quality has been associated with aging [13], shift work [2], sleep environments such as noise [14] and high room temperature [15], and exercise [16]. In Japan, the Ministry of Health, Labour and Welfare published Sleep Guidelines for Health Promotion [1], which were aimed at promoting good sleep quality and preventing lifestyle-related diseases. Though the important items related to sleep and exercise are highlighted, few items about dietary behavior are recommended.

Dietary intake may be one of the factors related to sleep quality, because previous studies have reported that poor sleep quality is associated with lower intakes of vegetables and fish, and higher intakes of confectioneries [17] and sugar-sweetened beverages [17,18]. Furthermore, a cross-sectional study in a large Japanese population indicated that good sleep quality is associated with better dietary quality, such as high intakes of vegetables, potatoes, mushrooms, seaweeds, eggs, and soy products [19]. Two methods are used to evaluate dietary quality. One is a posteriori approach by principal component analysis, and the other is a priori approach by a structured questionnaire about knowledge of dietary guidelines and nutrition [20]. A previous study using principal component analysis examined the association between a healthy dietary pattern and sleep quality [19]. However, because principal component analysis extracts dietary patterns in a specific study population, this method may result in the changes of elements for dietary patterns when the study population is different. Therefore, it should be noted that daily dietary quality evaluated by a pre-defined method leads to more robust results for daily dietary quality that do not depend on the study population than that by a posteriori approach.

Recent studies have been conducted using pre-defined methods, and some of the studies have indicated dietary patterns for an assessment of dietary quality based on adherence to the Japanese Food Guide Spinning Top [21,22,23,24]. The Japanese Food Guide Spinning Top was developed to help people implement the Dietary Guidelines for Japanese. It guides people as to what kinds and how much food they should eat each day to promote health [25]. The Japanese Food Guide Spinning Top consists of the following food categories: grain dishes (rice, bread, noodles, etc.), vegetable dishes (vegetables, mushrooms, potatoes, and seaweed), fish and meat dishes (meat, fish, eggs, soybeans, etc.), milk (milk and milk products), fruits, confectioneries, sugar-sweetened beverages, and alcoholic beverages. The order of the food groups is given by the size of the recommended daily servings. Combining the food groups daily is recommended. The Japanese Food Guide Spinning Top is a useful tool for assessing the dietary quality of Japanese people. Previous studies that have evaluated the degree of adherence to the Japanese Food Guide Spinning Top (Food Guide score) have reported associations between the Food Guide score and the risk of total mortality [23], metabolic risk factors [24], and depressive symptoms [22]. However, any association of sleep quality with the Food Guide score (i.e., balanced dietary intake) has not been determined. Therefore, in this study, our purpose was to elucidate the association between the Food Guide score and sleep quality in Japanese college students. Our hypothesis was that a well-balanced diet would be associated with sleep quality in young adults.

## 2. Methods 

### 2.1. Study Participants

This cross-sectional study was conducted between October and November 2017 in a population consisting of college students in the eastern part of Gunma Prefecture. Of the 216 participants who took lectures related to food, 175 participated in the survey (response rate: 81%). On the day of the survey, all participants provided written informed consent. This study was conducted according to the guidelines laid down in the Declaration of Helsinki and all procedures involving human subjects/patients were approved by the Faculty of Life Sciences, Department of Food and Environmental Sciences, Department of Comprehensive Informatics, and Faculty of Science and Technology, Toyo University (Approval No. TU2016-012). 

### 2.2. Assessment

A self-administered questionnaire, which was distributed to participants, included demographic characteristics, lifestyle habits, sleep quality, and food consumption. The completed questionnaire was returned on the same day it was distributed, and participants with mistakes or inconsistencies in their questionnaire responses were followed-up by well-trained researchers. Demographic characteristics (age, sex, body mass index (BMI), residential status (alone or other), self-reported current medical history and past medical history (yes or no), current drinker (yes or no) and smoker (yes or no)) were based on the Center for Epidemiologic Studies Depression Scale (CES-D) [26], which was developed for assessing the level of depressive symptoms in the general population; and a Japanese version of the Horne-Östberg Morningness-Eveningness Questionnaire (MEQ) for assessing self-rated morningness/eveningness preference were included in the survey [27,28]. BMI was calculated on the basis of self-reported height and weight (weight/height^2^ (kg/m^2^)). The CES-D is a 20-item measure that assesses the frequency of symptoms during the past week on a 0–3 Likert scale (“rarely or none of the time” to “most or all of the time”). The higher the CES-D score, the greater the tendency for depression. The MEQ is a 19 item measure, in which a lower MEQ score indicates greater eveningness [27] and has been associated with the delayed phase of the biological circadian rhythm (cortisol levels [29], body temperature [30,31]) and the sleep-wake cycle on free days [32].

For the assessment of sleep quality, the Japanese version of Pittsburgh Sleep Quality Index (PSQI) was used [33]. The PSQI is a 19-item self-rated measure that assesses sleep quality during the last month (e.g., bed time, sleep latency, and awakening time), according to 7 dimensions: sleep quality, sleep latency, sleep duration, habitual sleep efficiency, sleep disturbances, use of sleep medication, and daytime dysfunction. The scores from these 7 dimensions are added together, creating a global score (PSQI score). A PSQI score of ≥6 is considered as sleep disturbance [34,35]. The PSQI is among the most frequently used measures for assessing the level of sleep quality and is well-validated: the sensitivity, specificity, and Cronbach-alpha coefficient were 89.6%, 86.5%, and 0.77, respectively [9].

Habitual dietary intake over the previous 1 month was evaluated using a self-administered diet history questionnaire (DHQ) [36]. The questionnaire assesses eating behavior, frequency and amount of 110 foods consumed, frequency and amount of grain dishes consumed, and frequency and amount of other foods and dietary supplements consumed per day. Energy intakes derived from the DHQ have been reported to be moderately correlated with energy expenditures calculated by the double-labelled water method (correlation coefficient: 0.42 for men, 0.37 for women) [37]. Furthermore, the median correlation coefficient between the DHQ and the results obtained by the 16-day dietary record was 0.44 (range: 0.14–0.82) for men, 0.43 (range: −0.09–0.77) for women [36].

The Food Guide score was calculated from 7 categories of the Japanese Food Guide Spinning Top and was based on the results from the DHQ, according to previous studies [21,23,24]. Details of the categories used for calculating the Food Guide score are shown in Table S1. Servings of grain dishes, vegetable dishes, fish and meat dishes, milk and milk products, and fruits were calculated. One serving of a grain dish is composed of about 40 g carbohydrates. In one serving of a vegetable dish, the main ingredient weighs about 70 g. One serving of a fish and meat dish contains about 6 g protein, and one serving of milk and milk products contains about 100 mg calcium. In one serving of fruits, the main ingredient weighs about 100 g. The recommended amount of servings for each category (grain, vegetable, fish and meat, milk and milk products, fruits) and the recommended total energy intake are specified according to sex, age, and level of physical activity, whereas the recommended amount of energy intake from confectioneries, sugar-sweetened beverages, and alcoholic beverages is less than 200 kcal/day for everyone. If an individual consumed less than the recommended amount of servings or energy, the score was calculated with the following formula: 10 × (the consumed amount of servings or energy)/(the lower limit of the recommended amount). If an individual consumed more than the recommended amount of servings or energy, the score was calculated with the following formula: 10 － 10 × ((the consumed amount of servings or energy) － (the upper limit of the recommended amount))/(the upper limit of the recommended amount). All group scores were summed to provide the overall Food Guide score, which ranged from 0 to 70 [23]. A higher score is more likely to show diet adherence to the Japanese Food Guide Spinning Top and reflect the intake of a well-balanced diet.

### 2.3. Statistical Analysis

Of the 175 participants, we excluded participants who did not complete the PSQI, who reported extreme energy intake (lower than 500 kcal and more than 3500 kcal/day for women, lower than 800 kcal and more than 4000 kcal/day for men [38]), and who belonged to sport clubs. Therefore, a total of 155 participants were included in our statistical analysis. Data are presented as the mean ± standard deviation for continuous variables, as the number (%) for categorical variables, and as the odds ratio (OR) (95% confidence interval (CI)). Intakes of nutrients and food groups obtained from the DHQ were adjusted according to total energy intake using a residual method [39]. Because the Food Guide score was equally distributed among men and women, all participants were separated into tertiles (T1 to T3) according to the Food Guide score. The score of the T1 group (*n* = 57) ranged from 22 to 37 points, that of the T2 group (*n* = 47) from 38 to 43 points, and that of the T3 group (*n* = 51) from 44 to 62 points. The trend test or the Jonckheere-Terpstra test, χ^2^ test, and Goodman-Kruskal’s γ coefficient was used for continuous variables, categorical variables, and ordinal-scale variables, respectively. In the multivariate logistic regression analyses, the ordinal numbers 0–2 were assigned to the 3 categories of each Food Guide score to assess trend association. Model 1 was adjusted for age (continuous, in years), sex (0 = men, 1 = women), BMI (continuous, kg/m^2)^, residential status (0 = alone, 1 = other), current drinker (0 = no, 1 = yes), and current smoker (0 = no, 1 = yes). Model 2 was additionally adjusted for energy intakes (continuous, kcal) [19], CES-D scores (continuous, points) [9,17], MEQ score (1 = evening type, 2 = intermediate, or 3 = morning type) [40], and physical activity level (PAL) (range 1–4, 1 = sedentary, 2 = moderately, 3 = active, 4 = very active) [41]. Because depressive symptoms are associated with both dietary habits [42] and sleep symptoms [43], we used the CES-D score as a covariate in the multivariate adjusted model. Moreover, a test for linear trends was conducted by using the Food Guide score as a continuous variable in the logistic regression models. A *p* value <0.05 was considered statistically significant using two-tailed tests. All analyses were performed using IBM SPSS Statistics version 24.0 (Japan IBM Co., Ltd., Tokyo, Japan).

## 3. Results

Table 1 shows the associations between the Food Guide score and demographic characteristics. In this study, 77 women and 78 men were included in the analysis, and the mean age was 20 years old. No significant association (*p* > 0.05) was found between the Food Guide score and age, height, weight, BMI, sex, residential status, current smoker, current medical history, or past medical history. However, a significant association was found between the Food Guide score and current drinker (*p* < 0.05). Individuals with higher Food Guide scores were less likely to be a current drinker.

Table 2 shows a specific Food Guide score (i.e., scores for total energy, grain dishes, vegetables dishes, fish and meat dishes, milk and milk products, fruits, energy from confectioneries, sugar-sweetened beverages, and alcoholic beverages), and intakes of nutrients and food groups. A higher Food Guide score was significantly associated with higher scores for all the specific items, such as total energy, grain dishes, vegetables dishes, fish and meat dishes, milk and milk products, fruits, energy from confectioneries, sugar-sweetened beverages, and alcoholic beverages. A higher Food Guide score was significantly associated with lower or higher intakes of fat and carbohydrate. Regarding food group intakes, a higher Food Guide score was significantly associated with higher intakes of grains, green and yellow vegetables, non-green yellow vegetables, fruits, mushrooms, seaweeds, and meats, and with lower intakes of confectioneries, sugar-sweetened beverages, and alcoholic beverages (*p* < 0.05).

The associations between the Food Guide score and sleep quality are shown in Table 3. Individuals with a higher Food Guide score had a significantly higher prevalence of a good sleep score and a higher MEQ score (*p* < 0.05). No significant association (*p* > 0.05) was found between the Food Guide score and the time of retiring and awakening, sleep duration, or the midpoint of sleep timing.

**Figure 1:**
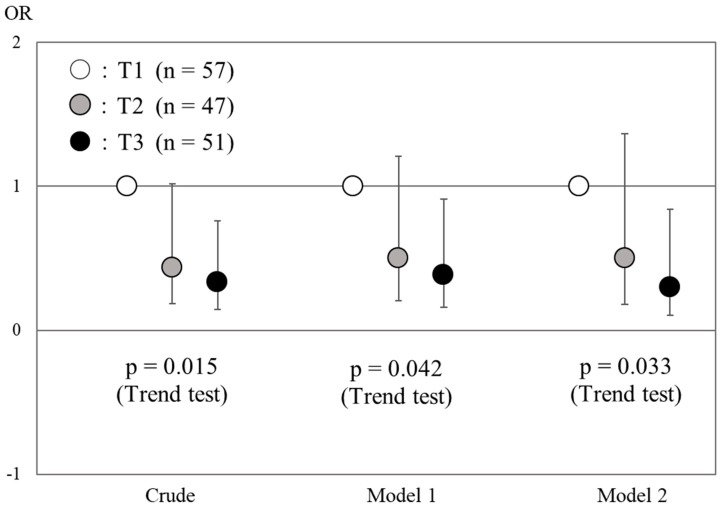
Association between the Food Guide score and sleep quality in multivariate logistic regression. ●: T1 group, ●: T2 group, ●: T3 group. T1, lowest tertile of the Food Guide score; T2, middle tertile of the Food Guide score; T3, highest tertile of the Food Guide score. OR, Odds ratio. Model 1 adjusted for age, sex, body mass index (BMI), current drinker, current smoker, and residential status. Model 2 adjusted for model 1 plus the Center for Epidemiologic Studies Depression Scale (CES-D), Morningness-Eveningness Questionnaire score (MEQ score), Physical Activity Level (PAL), and total energy intake.

In multivariate logistic regression, an association between the Food Guide score and sleep quality was confirmed with covariate adjustment (Figure 1). Univariate analysis showed that the OR (95% CIs) for the highest tertile versus the lowest was 0.33 (0.15–1.76) (*p* for trend = 0.015). In addition, after adjustment for age, sex, BMI, current drinker, current smoker, and residential status, the ORs (95% CI) were 0.50 (0.21–1.21) and 0.38 (0.16–0.91) for the T2 and T3 groups, respectively (*p* for trend = 0.042). Moreover, after adjustment for model 1 plus the CES-D score, MEQ score, PALs, and energy intakes, the significant association between the Food Guide score and sleep quality did not disappear (*p* for trend = 0.033). On the other hand, when we used the PSQI score as a continuous variable in model 3, there was no statistically significant association between the Food Guide score and the PSQI score (*p* = 0.167). In addition, because a significant association was found between the Food Guide score and the MEQ score (Table 3), we confirmed the additional analysis using the MEQ score and the Food Guide score as a dependent and independent variable, respectively. As a result, a higher Food Guide score was also significantly associated with a higher MEQ score (*p* = 0.026).

## 4. Discussion

Our cross-sectional study examined the association between the Food Guide score, which was based on adherence to the Japanese Food Guide Spinning Top, and sleep quality in Japanese college students. As a result, a higher Food Guide score was significantly associated with higher scores for all of the specific items (i.e., total energy, grain dishes, vegetable dishes, fish and meat dishes, milk and milk products, fruits, and energy from confectioneries, sugar-sweetened beverages, and alcoholic beverages). A higher Food Guide score was significantly associated with lower and higher intakes of fat and carbohydrate, respectively. Regarding food group intakes, a higher Food Guide score was significantly associated with higher intakes of grains, green and yellow vegetables, non-green and yellow vegetables, fruits, mushrooms, seaweeds, and meats, and with lower intakes of confectioneries, sugar-sweetened beverages, and alcoholic beverages (*p* < 0.05). A significant inverse association was found between the Food Guide score and the PSQI score. Furthermore, the multivariate-adjusted model revealed that the ORs (95% CI) of the T2 and T3 groups were 0.50 (0.18–1.37) and 0.30 (0.11–0.84), respectively (*p* for trend = 0.033). These results suggest that a higher Food Guide score indicates a well-balanced dietary intake characterized by the consumption of meats, fish, eggs and soy products, milk and milk products, and fruits, and may be associated with better sleep quality.

The present study shows that a higher Food Guide score was associated with better sleep quality. A previous cross-sectional study in 3129 women aged 34–65 years indicated that a higher PSQI score was significantly associated with lower intakes of vegetables and fish and higher intake of confectioneries [17]. Another cross-sectional study with a principal component analysis assessed a “healthy pattern” characterized by dietary intake, such as intakes of vegetables, mushrooms, potatoes, seaweeds, soybean products, and eggs, and showed that a higher score for the healthy pattern was associated with a decreased prevalence of difficulty falling asleep at least once a week [19]. Moreover, an interventional trial study in 29 patients aged 20–55 years examined the effect of increased intake of fruits for 4 weeks on self-rated sleep quality, and indicated that dietary intervention with increased fruit intake resulted in a significant decrease in the PSQI score, increased sleep efficiency, increased sleep duration, and decreased sleep latency compared with before the intervention [44]. Therefore, these results are consistent with the results of the present study. Our study used a priori approach with a structured questionnaire to assess daily dietary intake, which does not depend on the study population; to our knowledge, this is the first study to identify the association between higher habitual dietary quality, which was assessed using a comprehensive approach (i.e., adherence to the Japanese Food Guide Spinning Top), and better sleep quality in Japanese college students. However, because our significant result weakened when the PSQI score was used as a continuous variable, further large population-based studies are needed to elucidate the causality between dietary quality and objective and subjective sleep quality using experimental and longitudinal studies.

Though the underlying mechanisms could not be elucidated in this study, the key potential factors, which may be related to sleep quality, are combinations of various nutrients, such as protein, carbohydrate, and amino acids. For example, an epidemiological study of 4435 men and women examined the association between the percentage of energy intake from protein and subjective sleep quality, and found that individuals whose percentage of energy intake from protein was 16% or more had a significantly lower risk of poor sleep quality than those with an intake of 16% or less [45]. Because the amino acid l-tryptophan (Trp) [46,47] supplied by dietary protein is a precursor of serotonin and melatonin [47,48], and secretion of serotonin and melatonin regulates the sleep-wake cycle [49], intake of Trp may mediate the association between the Food Guide score and sleep quality. In contrast, a previous study showed that carbohydrate and protein uptake rates are involved in the transport of Trp from the blood to the brain [50], suggesting the necessity for intakes of carbohydrate as well as Trp. Intake of milk and milk products [51] and magnesium [52] has been reported to be associated with sleep quality. A higher Food Guide score was significantly associated with a higher intake of grain dishes, high intake of fish and meat dishes, and milk and milk products, and a higher Food Guide score was also significantly associated with intake of various nutrients, such as magnesium, iron, vitamin B1, vitamin C, dietary fiber, zinc, vitamin B6, folate, and vitamin K (data not shown); therefore, a well-balanced diet consisting of various foods may have a beneficial role in maintaining good sleep quality. Consequently, further details of the effect of a balanced diet and the impact of specific foods and nutrients on sleep quality should be investigated in a prospective and experimental study design.

The National Health and Nutrition Survey in Japan reported that the percentage of people in their 20s who have bad sleep quality was lower than that in those aged 30–59 [53]. However, the eveningness preference peaked in those aged in their 20s [54], and was positively associated with social jet lag (i.e., misalignment between social and biological time) [55], which is a potential risk of poorer sleep quality. Because approximately 60% of participants in our study had a PSQI score greater than the cutoff value (6 points), and this result was consistent with a previous study [6], young adults with sleep problems should not be overlooked. On the other hand, the percentage of adults in their 20s skipping breakfast was 30.6% for males and 23.6% for females, and was higher than that in other age groups [56]. A previous study of Japanese college students indicated that habit of skipping meals was associated with poorer dietary quality. These findings suggest the important role of well-balanced dietary intake for maintaining sleep quality. Therefore, our findings shed light on the need for nutritional support for sleep hygiene in young adults.

The Food Guide score was significantly associated with intakes of grains, green and yellow vegetables, non-green and yellow vegetables, fruits, mushrooms, seaweeds, and milk and milk products, because the Food Guide score is calculated from the adherence to the Japanese Food Guide Spinning Top. This result is consistent with a previous cross-sectional study in terms of the significant associations between diet quality score and nutrient and food group intakes based on the DHQ [22]. In addition, another study reported that a higher diet quality score was significantly associated with higher intakes of cereals, vegetables, meat, fish, milk and milk products, and fruits, and lower energy intakes from confectioneries, sugar-sweetened beverages, and alcoholic beverages [24]. Therefore, the Food Guide score calculated by a priori approach (i.e., a structured method) may reflect balanced dietary intake by consuming diverse foods in daily life.

The Food Guide scores ranged from 0 to 10 for the specific items, such as grain dishes and vegetable dishes, fish and meat dishes, milk and milk products, fruits, energy from confectioneries, sugar-sweetened beverages, alcoholic beverages, and total energy, and a total score from 0 to 70 was calculated. Therefore, there is a possibility that the total score would be high, even if the score for specific items was low. As a result, the use of the Food Guide score may lead to misclassification of balanced dietary intake in daily life. However, when we confirmed whether the Food Guide score was positively associated with the scores of the specific items, a significant association between the Food Guide score and the score of 7 items was found, indicating that a higher Food Guide score is not consistent with the score derived from unbalanced dietary intake. In a previous study of 42,970 middle-aged and older adults (45 to 75 years old), the mean Food Guide score, which was calculated using the same method as our study, was 47.4 points [23], while the score in the present study was 40.9 points in young adults. Therefore, the adherence to the Japanese Food Guide Spinning Top is considered to be higher with age, and college students may be more likely to have a lower score than other generations. The strength of the association between the Food Guide score and sleep quality may be underestimated in this population because of the narrow distribution of the Food Guide score. Further studies with a wide range of scores (e.g., various generations) are needed.

Although the strongest stimulus for entraining the biological clock to the sleep-wake cycle and/or external day-night cycle (i.e., phase resetting effect) is the light signal, non-photic factors, such as eating behaviors, can also have a direct or indirect effect on the circadian system in animal [57] and human studies [58]. Therefore, dietary behavior may be one of factors which could shift diurnal preference (i.e., the degree to which people prefer to be active in the morning or the evening) from eveningness to morningness. However, with regards to the significant association between the Food Guide score (i.e., quality of dietary intake) and the MEQ score in our study, it should be noted that most previous studies focused on the timing of eating rather than the quality of the diet, and other studies explored the association using the MEQ score as an exposure factor [59]. Therefore, further studies are needed to clarify causality and to indicate the potential possibility of the phase resetting effect by the habitual balanced dietary intakes.

Several potential limitations should be addressed. First, our study was a cross-sectional study in a limited population, which may lead to the over- or under-estimation of point estimates caused by sampling bias. Therefore, the generalization of our results may be limited because the samples consisted of a limited and small number of college students. Second, variables such as dietary intake, which is used for the Food Guide score, and sleep quality were self-reported. Third, there may be potential residual confounding by unmeasured variables, such as skipping meals [17], time of meals, napping [8], physical activities [60,61], and sleeping environment [14]. Finally, the score of the specific 7 items was summed without being weighted. A weighted score for a particular food item may better reflect the dietary quality of young adults.

## 5. Conclusions

In this study, a significant association between well-balanced dietary intake and self-rated sleep quality was elucidated in Japanese college students. Longitudinal studies in various generations and experimental studies using objective measures of sleep quality are necessary to clarify the causality between well-balanced dietary intake and sleep quality.

## Figures and Tables

**Table 1:** Association between the Food Guide score and demographic characteristics.

	T1		T2		T3		*p* Value
	(*n* = 57)		(*n* = 47)		(*n* = 51)		
Age *(years)	20.0	0.7	20.0	0.8	20.0	0.8	0.974
Height *(cm)	165.8	7.7	166.0	9.3	163.8	10.0	0.255
Weight *(kg)	57.8	8.7	58.2	11.5	57.2	11.9	0.615
BMI *(kg/m^2^)	21.0	2.5	21.0	3.2	21.2	3.0	0.603
Sex ^†^							
Men	27	47.4	25	53.2	26	51.0	0.692
Woman	30	52.6	22	46.8	25	49.0	
Residential Status ^†^							
Living Alone	22	38.6	20	42.6	18	35.3	0.762
Other	35	61.4	27	57.4	33	64.7	
Current Drinker ^‡^							
No	27	47.4	32	68.1	39	76.5	0.001
Yes	30	52.6	15	31.9	12	23.5	
Current Smoker ^‡^							
No	51	89.5	45	95.7	48	94.1	0.350
Yes	6	10.5	2	4.3	3	5.9	
Current Medical History ^‡^							
No	53	94.6	45	97.8	47	92.2	0.634
Yes	3	5.4	1	2.2	4	7.8	
Past Medical History ^‡^							
No	49	89.1	40	87.0	41	85.4	0.574
Yes	6	10.9	6	13.0	7	14.6	

Values are presented as mean ± standard deviation for continuous variables or as a number (%) for categorical variables. BMI, body mass index, * Jonckheere-Terpstra test, ^†^ χ^2^ test, ^‡^ Goodman-Kruskal’s γ.

**Table 2:** Association between the Food Guide score, specific Food Guide score, and intake of nutrients and food groups.

	T1		T2		T3		*p* Value
	(*n* = 57)		(*n* = 47)		(*n* = 51)		
Total Food Guide Score ^‡^	33.1	3.4	41.2	1.4	49.3	4.5	<0.001
Total Energy (points) ^†,§^	8.1	1.8	8.6	1.4	9.4	0.9	<0.001
Grain Dishes (points) ^†,§^	7.6	1.8	8.4	1.5	8.7	1.2	<0.001
Vegetable Dishes (points) ^†,§^	4.3	2.9	5.1	2.7	6.1	2.7	0.001
Fish and Meat Dishes (points) ^†,§^	5.3	4.0	6.9	3.4	7.0	3.4	0.003
Milk and Milk Products (points) ^†,§^	2.7	2.5	3.6	2.8	5.5	3.1	<0.001
Fruits (points) ^†,§^	3.2	3.2	3.7	3.2	5.5	3.4	<0.001
Energy from confectioneries, sugar-sweetened beverages and alcoholic beverages (points) ^†,§^	2.4	3.7	5.3	4.3	7.5	3.4	<0.001
Energy Intake	2037	768	1892	550	1832	338	0.071
Nutrient Intake *^,‡^							
Protein (g)	62.1	14.2	64.9	11.2	66.5	10.9	0.094
Fat (g)	68.5	18.6	64.9	14.0	62.2	13.0	0.036
Carbohydrate (g)	265.5	50.8	272.2	34.2	282.3	34.4	0.025
Food Group Intake *							
Grains (g) ^‡^	420.1	135.5	465.2	132.3	499.3	115.4	0.001
Potato (g) ^‡^	25.4	25.8	25.3	19.9	24.9	18.2	0.395
Nuts and Seeds (g) ^‡^	0.8	1.5	0.7	1.5	2.2	4.3	0.225
Green Yellow Vegetables (g) ^‡^	44.5	44.6	60.3	36.3	109.5	96.5	<0.001
Non Green Yellow Vegetables (g) ^‡^	70.3	51.1	85.7	56.1	97.7	62.0	0.008
Mushrooms (g) ^‡^	4.6	6.6	9.1	9.1	8.9	11.5	0.015
Seaweed (g) ^‡^	5.8	8.4	10.3	12.8	9.5	10.7	0.005
Fish and Shellfish (g) ^‡^	39.2	40.2	42.6	28.4	49.5	28.4	0.113
Meats (g) ^‡^	91.6	52.8	94.4	43.0	85.8	36.6	0.768
Eggs (g) ^‡^	37.5	44.9	34.6	21.5	34.2	25.3	0.498
Beans (g) ^‡^	37.4	33.4	40.2	27.6	50.1	46.1	0.062
Milk and Milk Products (g) ^‡^	101.8	144.4	124.2	144.6	128.9	88.0	0.007
Fruits (g) ^‡^	61.7	99.4	100.9	86.2	158.4	125.2	<0.001
Confectioneries (g) ^‡^	107.2	57.7	86.2	50.7	64.4	30.8	<0.001
Sugar-Sweetened Beverages (g) ^‡^	843.0	546.1	781.6	686.9	775.3	478.9	0.753
Alcoholic Beverages (g) ^‡^	82.4	128.5	56.3	121.3	18.5	31.7	0.001

Values are presented as mean ± standard deviation for continuous variables. ^‡^ Jonckheere-Terpstra test, ^†^ Trend test, * Data were adjusted for energy intake using a residuals method. ^§^ Possible score ranging from 0 to 10.

**Table 3:** Association between the Food Guide score and sleep quality.

		T1		T2		T3		*p* Value
		(*n* = 57)		(*n* = 47)		(*n* = 51)		
Awaking Time * (h:min)		7:46	1:10	7:26	1:06	7:31	1:12	0.152
Retiring Time * (h:min)		24:08	1:04	23:54	0:56	23:44	1:02	0.087
Sleep Duration * (h:min)		7:38	0:56	7:32	1:08	7:47	1:00	0.493
Midpoint of Sleep *^,§^ (h:min)		3:49	0:30	3:43	0:33	3:56	0:29	0.236
PSQI Score *^,†^ (points)		7.3	2.5	6.1	2.2	6.1	2.5	0.003
	<6	13	22.8	19	40.4	24	47.1	0.006
	6≤	44	77.2	28	59.6	27	52.9	
MEQ Score ^‡^ (points)		47.3	7.1	49.6	7.6	50.4	7.0	0.025

Values are presented as mean ± standard deviation for continuous variables or as number (%) for categorical variables. PSQI, Pittsburgh Sleep Quality Index; MEQ, Morningness-Eveningness Questionnaire; * Jonckheere-Terpstra test, ^†^ Goodman-Kruskal’s γ, ^‡^ Trend test, ^§^ The midpoint of sleep was calculated as the halfway point between bedtime and rise time.

## Supplementary Materials

The following are available online at http://www.mdpi.com/2072-6643/10/12/1996/s1, Table S1: Details of the categories used for calculating the Food Guide score.
