# Supplementary material for: Association between Adherence to the Japanese Food Guide Spinning Top and Sleep Quality in College Students
Source: Nutrients. 2018 Dec 16;10(12):1996. doi: 10.3390/nu10121996 (PMC6316252; doi:10.3390/nu10121996)
Supplement: Supplementary file 1 [file nutrients-10-01996-s001.pdf]

Table S1. Details of the categories used for calculating the Food Guide score

**Grain dishes**	
Rice and rice products	White rice, barley rice, germ rice, half threshing, 70% threshing brown rice
Noodle and pasta	Buckwheat, wheat noodles, thin wheat noodles, instant noodles, cup noodles, other Chinese noodles, spaghetti and pasta
Bread and other	Bread, butter rolls, croissant, pizza, okonomiyaki, corn flakes
**Vegetable dishes**	
Green and yellow vegetables	Carrot, pumpkin, tomato, green pepper, broccoli, green-leafy vegetables
Other vegetables	Cabbage, cucumber, lettuce, Chinese cabbage, sprouts, radish, onion, cauliflower, eggplant, burdock, lotus root, fried potato, potato, sweet potatoes, taro, konjac, seaweed, laver, peanuts, nuts, vegetable juice, tomato juice
Pickles	Pickled ume, pickled vegetables and pickled products
Mushrooms	Mushrooms
**Fish and meat dishes**	
Meats	Ground meat (beef and/or pork), chicken, pork, beef, liver, ham or sausage, bacon
Fish	Dried fish, small fish with bones, tuna (oily), broiled eel, fish with white meat, fish with blue skin, fish with red meat, fish paste products, shrimp, squid, octopus, oysters, other shellfish, fish eggs, salted fish roe, foods boiled down in soy sauce (tsukudani)
Egg	Eggs
Soy products	Tofu, tofu products as ganmodoki, natto, cooked soybean, soybean milk miso (except for miso soup), miso (miso in miso soup)
**Milk and milk products**	
Milk	Milk (normal milk), milk (low fat), skim milk
Yogurt	Yogurt (sweet and/or sugarless and/or low-sugar)
Cheese and other milk products	Cheese, cottage cheese, cream in coffee
**Fruits**	
Fresh fruits	Oranges, bananas, apples, strawberries, grapes, peaches, pear, date plum, kiwi fruit, melon, watermelon
Other fruits	Dried grapes, canned fruits, 100% fruit juice
**Energy from confectioneries, sugar-sweetened beverages, and alcoholic beverages**	
Confectioneries	Potato chips, snack confectioneries, Japanese confectioneries (including bean jam), Japanese confectioneries (not including bean jam), confectioneries, cookies, chocolate,

	rice cracker, candy and/or caramel and/or gum, jelly, sweet buns, pancakes, doughnut,<br>ice cream with milk-solids content of 3% or greater,<br>ice cream with milk-solids content of 15% or greater
Alcoholic drinks	Beer, sake, shochu, white liquor highball, whiskey and wine.
Non-alcoholic drinks	Coffee, milk beverages, 50% fruit juice, cocoa, sugar-sweetened beverages, non-calorie sugar-sweetened beverages, green tea, tea, oolong tea (including other Chinese teas), revitalizer.
**Total energy intake**	
